# A Virtual Navigation Training Promotes the Remapping of Space in Allocentric Coordinates: Evidence From Behavioral and Neuroimaging Data

**DOI:** 10.3389/fnhum.2022.693968

**Published:** 2022-04-05

**Authors:** Katiuscia Sacco, Irene Ronga, Pasqualina Perna, Alessandro Cicerale, Elena Del Fante, Pietro Sarasso, Giuliano Carlo Geminiani

**Affiliations:** BIP (BraIn Plasticity and Behavior Changes) Research Group, Department of Psychology, University of Turin, Turin, Italy

**Keywords:** memory, virtual reality training, spatial orientation, learning, brain plasticity

## Abstract

Allocentric space representations demonstrated to be crucial to improve visuo-spatial skills, pivotal in every-day life activities and for the development and maintenance of other cognitive abilities, such as memory and reasoning. Here, we present a series of three different experiments: Experiment 1, Discovery sample (23 young male participants); Experiment 2, Neuroimaging and replicating sample (23 young male participants); and Experiment 3 (14 young male participants). In the experiments, we investigated whether virtual navigation stimulates the ability to form spatial allocentric representations. With this aim, we used a novel 3D videogame (*MindTheCity!*), focused on the navigation of a virtual town. We verified whether playing at *MindTheCity!* enhanced the performance on spatial representational tasks (pointing to a specific location in space) and on a spatial memory test (asking participant to remember the location of specific objects). Furthermore, to uncover the neural mechanisms underlying the observed effects, we performed a preliminary fMRI investigation before and after the training with *MindTheCity!*. Results show that our virtual training enhances the ability to form allocentric representations and spatial memory (Experiment 1). Experiments 2 and 3 confirmed the behavioral results of Experiment 1. Furthermore, our preliminary neuroimaging and behavioral results suggest that the training activates brain circuits involved in higher-order mechanisms of information encoding, triggering the activation of broader cognitive processes and reducing the working load on memory circuits (Experiments 2 and 3).

## Introduction

Being able to navigate the environment we live in is crucial for survival. Organisms not only have to explore the environment, but they also need to *remember* previous navigation attempts to establish safe and successful interactions with the outer world. Not surprisingly, it has been proposed that memory as a whole can be viewed as a form of “navigation in mental space,” grounded on the same neural algorithms guiding real space exploration (Buzsáki and Moser, 2013). Classical memory aids exploit visuo-spatial associative techniques, e.g., method of *loci* or *memory palaces*, where people mentally navigate imagined structures to recall information (Yates, 1966). The use of spatial mnemonic strategies has been demonstrated to enhance (even non-spatial) memory performances (Worthen and Hunt, 2011), both in superior memorizers and control participants (Dresler et al., 2017). Interestingly, Maguire et al. (2003) found that the brain networks involved in spatial learning strategies largely overlapped with the brain areas devoted to actual spatial navigation (such as the retrosplenial cortex—see also Maguire et al., 1998). In line with this results, visuo-spatial skills are considered pivotal in supporting the development and maintenance of other cognitive abilities, such as memory, mental imagery, and reasoning (Bellmund et al., 2018). Therefore, it is possible that spatial navigational trainings might improve spatial and non-spatial memorization abilities, by the enhancement of shared neural mechanisms. More specifically, Maguire et al. (2003) found that, during a memorization task, the medial parietal areas, the retrosplenial cortex, and the right posterior hippocampus showed greater activations in the group of super memorizers as compared to the control group, thus suggesting their implication in the observed enhanced memory encoding. Interestingly, these areas are shared with the brain network used for actual navigation tasks in humans (Maguire et al., 1998; Sherrill et al., 2013; Patai et al., 2019) and in other mammals, such as rodents (Clark et al., 2018). It is worth nothing that the retrosplenial cortex is also connected with other areas involved in spatial navigation (Mitchell et al., 2018), most of all the parahippocampal regions (Epstein, 2008).

When we navigate a space, we build an *egocentric* representation of the surrounding environment, i.e., the memory trace of the sequence of elements and landmarks encountered along a route. However, through further explorations, we may also develop *allocentric* representations, capturing the spatial relationships between environmental elements, independently from individuals’ subjective perspective (Thorndyke and Hayes-Roth, 1982; Latini-Corazzini et al., 2010). Navigational strategies may rely on route (mostly based on landmark and path information) or survey (preserving the Euclidian relationships between locations in space) cognitive maps. Importantly, allocentric representations are suggested to be crucial in the formation of survey maps, since both are grounded on spatial relationships independently from individual specific position and covered path (Carelli et al., 2011). Previous studies suggested that the retrosplenial cortex is the region supporting the conversion of spatial information from an egocentric perspective to allocentric coordinates (Epstein et al., 2007, 2017; Cacucci et al., 2008; Vann et al., 2009; Eichenbaum and Cohen, 2014). This “re-coding” is crucial to improve visuo-spatial skills since, thanks to allocentric representation high level of plasticity, it allows spatial remapping when necessary. Not surprisingly, the ability to map the space in allocentric coordinates seems to progressively decline in healthy aging (Klencklen et al., 2012; Bates and Wolbers, 2014), and its deterioration represents an early clinical marker of prodromal forms of dementia, such as Mild Neurocognitive Disorder (MND), and Alzheimer’s Disease (AD) (Pengas et al., 2010; Serino et al., 2014; Marková et al., 2015; Coughlan et al., 2019). Potentiating the ability to shift from egocentric to allocentric representations may constitute a fundamental challenge to enhance learning mechanisms in young people, preventing visuo-spatial ability detriment in healthy elderly, and contrasting topographical disorientation in AD.

Virtual reality (from now on *VR*) is becoming rather popular in spatial exploration studies (Coughlan et al., 2019; König et al., 2020). First, compared to other spatial navigation trainings and tests (such as paper and pencil tasks), it represents a more ecological option. Its better adherence to real-life navigation makes VR a sensitive assessment tool for visuo-spatial ability impairments (Caglio et al., 2012; Plancher et al., 2012; Marková et al., 2015) as well as an effective rehabilitative training (Caglio et al., 2012; Faria et al., 2016; Smith, 2019). Furthermore, it allows safer and more controlled space explorations as compared to real-life ones, at the same time granting the opportunity to match virtual navigation with the measurement of behavioral and neural activity parameters (Serino et al., 2014).

**FIGURE 1:**
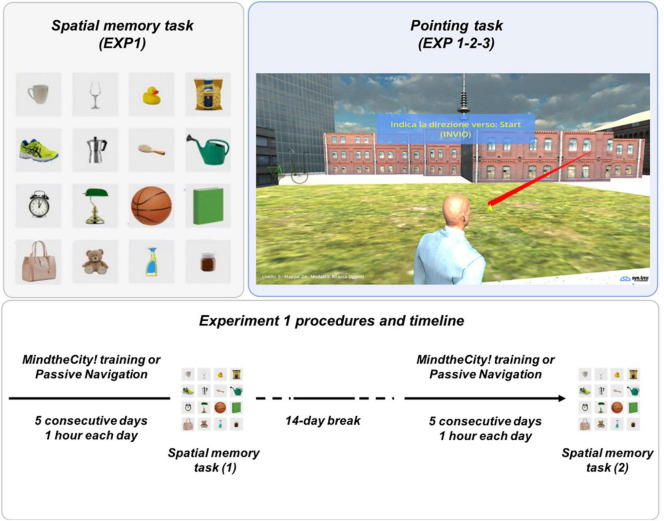
Experimental tasks (Experiments 1 and 2). *Spatial memory task **(top left panel)***: subjects were asked to remember the location of the objects presented in figure (Experiment 1). *Pointing task **(top right panel)***: in the videogame, subjects were asked to point to specific locations in the virtual space (Experiments 1-2-3). *Experiment 1 procedures and timeline **(bottom panel)***: the figure describes the experimental design of Experiment 1.

In the present study, in a series of two different experiments, we aimed at investigating the ability of a specific novel 3D videogame, *MindTheCity!*, in training players’ visuospatial skills and, more specifically, their abilities in building allocentric space representations from first-person perspective navigation. The VR environment represented a town that participants had to repeatedly explore in order to locate and retrieve specific objects. Importantly, the environment might be explored with the only help provided by visual information, since auditory cues were excluded from the game. In Experiment 1 (discovery sample), we investigated whether focused navigation (1 h for five consecutive days) in a virtual environment with few explicit *local landmarks* (such as highly distinguishable buildings) stimulates the ability to form allocentric representations, thus increasing performance on spatial representational tasks (such as pointing to a specific location in space—see also Figure 1). Furthermore, we verified whether the improvements driven by the virtual navigation task may be captured by a spatial memory test (i.e., asking participant to remember the location of specific objects placed in a rows-columns pattern—see also Figure 1), whose realization is known to be grounded on allocentric coordinates (Mou et al., 2008; Pasqualotto et al., 2013). However, results of Experiment 1 could not uncover the mechanisms underlying the possible observed effects. In other words, we could not disentangle whether the possible memorization enhancement is limited to specific improvements of visuo-spatial encoding, or whether it may generalize to other learning mechanisms. Therefore, in Experiment 2 (neuroimaging-replicating sample), we investigated whether results of Experiment 1 pointing task might be replicated by a second, independent sample. More importantly, through a preliminary fMRI investigation, we explored whether the allocentric navigation training produces brain changes, which may be observed in a classic associative memory encoding task (*paired associates*) even in the non-spatial verbal domain. Furthermore, in Experiment 3 we verified whether the training with *MindTheCity!* was able to elicit observable behavioral enhancements in a word-pair memorization task.

## Materials and Methods

### Participants

Healthy young male students from the University of Turin volunteered to take part in the experiments: 23 subjects (mean age: 25; *SD*: 2.93) participated in Experiment 1, other 23 subjects (mean age: 24.21; *SD*: 2.89) participated in Experiment 2, and other 14 healthy right-handed young male subjects (mean age: 25; *SD*: 3.56) participated in Experiment 3. Sample size of Experiments 1 and 2 was *a priori* determined according to a power analysis applied to the results of a pilot experiment (*N* = 5) testing the effect of *MindTheCity!* training on a directional pointing task (dependent variable: pointing angular errors). The experimental procedures were identical to those of Experiments 1 and 2. Thus, we computed the required sample numerosity to find a statistical difference between the angular errors made in the first half of the training vs. the errors made in the second half of the training, and to reach a power of 0.95 (power analysis results: effect size d = 0.8, sample size = 23, actual power = 0.96). We then targeted the desirable sample size (*N* = 23) in the Experiments 1 and 2.

Because behavioral studies have shown that there are gender differences in navigational task performances, a gender homogenous group (i.e., male only) was chosen (Astur et al., 1998). Only right-handed subjects were selected and the evaluation was made with the Edinburgh Handedness Inventory (Oldfield, 1971). Moreover, usual videogame users were excluded, especially for videogames in which the creation of mental maps of virtual environments is required for playing to avoid contamination effects on memory formation processes.

All participants were medication free with no history of psychiatric or neurological disorders. All participants gave their written informed consent to participate in the study, which conformed to the standards required by the Declaration of Helsinki and was approved by the Ethics Committee of the University of Turin (Prot. n. 121724—01/03/18). Participants were not compensated for taking part in the experiments.

### The Training

*MindTheCity!* is a 3D-videogame set in a fictional town (see Figure 1), developed using Unity.^1^ Graphics and the software for data collection were realized by *SynArea Consultants Srl* (Turin, Italy). MindTheCity! is divided into five districts, with progressively increasing dimensions (and therefore progressively increasing navigation difficulty), from the first to fifth. The town includes only few environmental landmarks: A central garden (the starting point for each district exploration), a church, a skyscraper, a telecommunication tower. All other buildings and streets present the very same characteristics, in order to encourage users to adopt a survey strategy of navigation and to create their own cognitive map of the environment (Ishikawa and Montello, 2006).

The main aim of the game is to complete a wayfinding task. Participants, while exploring the town with their virtual avatars (by pressing PC arrow keys), were asked to assemble five components of a bicycle (wheels, handlebars, pedals, rear frame and front frame), which were scattered around each district. In a first phase, participants had to explore the neighborhood freely in order to locate all bike components without collecting them (*free exploration phase*). In a second phase, participants were asked to remember where the previously identified components of the bike were located, in order to find the shortest path to retrieve them (*object search phase*). In this phase, anytime players collected one bike component, they had to perform a *pointing task* through their avatar (see Figure 1). They were requested to point an arrow toward the direction where they started the exploration (for the first retrieved component) or toward the previously retrieved bike component (from the second component on; see section “Assessment” for further explanations).

Two parallel versions of the game were designed (A and B) differing only in bike components positioning, in each district of the virtual town. This was done to exclude that training results might be related to a specific distribution of the objects within the virtual town, instead of the training itself. Each participant was presented with both versions of the game during the training. Version order (A–B; B–A) was counterbalanced across subjects, to avoid any sequence effects on results. All participants played *MindTheCity!* 1 h per day, for five consecutive days, in accordance with previous literature showing that this time is sufficient to improve spatial orientation (Kober et al., 2013; Claessen et al., 2016). During each session, at least two districts (of increasing dimension) were explored, with a 10–15 min break between them, for a total time of 50 min of training per day. In Experiment 1, the active training with Mindthecity! was contrasted with a passive navigation control condition, through a within-subject design (i.e., the involved participants performed both the active and the passive training, conditions’ order was counterbalanced across participants; see section “Experimental Procedures”).

### Assessment

#### Pointing Task (Experiments 1, 2, and 3)

As mentioned above (section “The Training”), during the training, participants were asked to point an arrow toward the direction where they started the exploration (for the first retrieved component) or toward the previously retrieved bike component (from the second component on). This pointing task aimed to verify whether participants were successful in representing the map of the district in allocentric coordinates. In immersive VR training, pointing tasks usually require the access of a mix of egocentric (to establish the position of the own body in the virtual space) and allocentric representations (to map the environment independently from their own point of view). However, in MindTheCity!, differently from an immersive virtual environment, the avatar is always visible as an external object during the pointing task (see Figure 1), thus likely decreasing the reliance on egocentric representations. For this reason, even though we cannot exclude that the egocentric representations formed during the avatar virtual navigation may still play a role in our pointing task, we believe that the realization of this exercise mainly relies on allocentric coordinates (for a fully selective, less ecological, test for allocentric coding, refer to Negen et al. (2018).

Degrees of angular error between the avatar’s pointing and targets’ actual positions were used as a performance measure and were recorded throughout the experiment. Importantly, the actual direction of the pointing was always retained in the computation of the angular error. During the game, no feedback of angular errors was given to participants.

#### Tracking Exploration Strategies (Experiment 1)

With the aim of further investigating whether and how *MindTheCity!* training might promote participants’ abilities to map the virtual environment, we tracked subjects’ navigational strategies during the training. As described above (section “The Training”), in the first free exploration phase, participants might freely explore the town to locate the bicycle components. In the second, object search phase, participants were asked to collect all the previously identified bicycle components. In Experiment 1, we tracked subjects’ navigation of the virtual environment (in districts 3 and 5, explored in days 2 and 4, and in days 3 and 5, respectively), recording the sequence of the located objects in the free exploration phase and of the collected objects in the search objects phase. Furthermore, we recorded the time spent to find the objects in both phases. If participants collected the objects in the same sequence and in a similar time between the free exploration and the object search phases, we should assume that they were employing a route strategy (mainly based on environmental landmark) to navigate the virtual environment, which would promote the adoption of the same path between exploration and object search. Conversely, if participants collected the objects in a different sequence and in a shorter time between the free exploration and the object search phases, the adoption of a survey strategy (mainly based on Euclidean relationship between different locations) appeared more likely, allowing them to select the most convenient path to cover to collect all the objects as quickly as possible. Importantly, the adoption of a survey strategy, as opposed to a route strategy, has been proposed to mainly rely on allocentric representations, which similarly to survey maps, are focused on the spatial relationships between objects (see Carelli et al., 2011; even though also egocentric representations may contribute to cognitive map realization). Therefore, the employ of a survey strategy would support the hypothesis that MindTheCity! training actively promotes the development of allocentric representations. Conversely, the adoption of a route strategy would indicate that participants mainly relied on path-based, egocentric information.

#### Spatial Memory Task (Experiment 1)

Following the training with MindTheCity! and following the control condition (passive navigation, see section “Experimental Procedures” below), participants were asked to perform a *spatial memory task* (i.e., a *spatial object-location memory task* (Postma et al., 2008; Zimmermann and Eschen, 2017), where subjects had to locate some objects in their correct locations in space and recording subjects’ responses), developed using Unity (see text footnote 1). Graphics and the software for stimulus presentation were realized by *SynArea Consultants Srl* (Turin, Italy). The visual stimuli employed in the paradigm were 16 everyday-life object images (elaborated using Photoshop), presented on a rows–columns pattern (4 × 4), composed of white square panels (all of identical dimensions). The background of the square panel was a fictional room wall (see Figure 1 Left panel). The task consisted of a simple memorization and recall test. Each session included four blocks, for a total duration of 36 min. Each block started with the Memorization Phase (duration: 2 min), where subjects were asked to pay attention to the spatial configuration of the objects with the aim of learning and remembering it. Following the Memorization Phase, subjects were asked to perform a distraction task (a pitch discrimination task similar to Sarasso et al., 2019; Sarasso et al., 2021, see also Sarasso et al., 2020) for 5 min, to avoid a rehearsal effect on subsequent memory performance. In the final Recall Phase, subjects were asked to place 10 (out of 16) objects in the exact position displayed in the Memorization Phase. Each object placement had to be completed in a maximum of 10 s. A green bar showed time progression toward the ending of the available time. The block (Memorization Phase, Distraction task, Recall Phase) was repeated four times, each time presenting a different random-generated object layout.

This task was used to assess whether participants’ visuospatial abilities were modulated by the realization of the training with MindTheCity! vs. Passive Navigation (see below section “Experimental Procedures”), and, more specifically, whether participants’ skills in forming allocentric spatial representations were affected. The present spatial memory task particularly promotes the use of allocentric representations. The objects to be remembered are positioned in a grid, thus encouraging the adoption of allocentric coordinates (Postma et al., 2008). Furthermore, the objects were all close to each other, rather than being spread in a large virtual environment (see Figure 1), thus resulting all in front of the participants, therefore making the use of egocentric coordinates unsuitable. For these reasons, although we cannot fully exclude that egocentric representations still affect participants’ performance, we believe that the realization of the task mainly relies on the successful employment of allocentric representations.

### Experiment 1 (Discovery Sample)

#### Experimental Procedures

Experiment 1 was conducted to measure the behavioral effects of *MindTheCity!* on spatial representations and memory. Participants were randomly assigned to the experimental condition—training with *MindTheCity!*—or to a control condition—Passive Navigation with *MindTheCity*! (i.e., passively observing an avatar exploring the same virtual town). Importantly, both conditions were carried out at the university laboratory, in presence of the experimenter. For five consecutive days (a first day of familiarization followed by 4 days of actual training), participants were asked either to play *MindTheCity!* (experimental condition) or to undergo the Passive Navigation (control condition). The order of these two conditions was counterbalanced across subjects. The two experimental conditions (i.e., active training with MindTheCity! vs. Passive navigation control condition) were separated by a break of 14 days. On the last day of *MindTheCity!* training and of Passive Navigation, participants received the post-training assessment on the spatial memory task specifically designed for the present study.

#### Data Analyses

##### Pointing Task

To evaluate subjects’ performance at *MindTheCity!*, angular errors in the pointing task were recorded. Single subject angular errors were averaged in order to obtain one single measure for each session. We therefore summed the measurements collected in the first half (days 2–3) and in the second half (days 4–5) of the training, respectively, thus resulting in two distinct values for each subject. Paired-samples *t*-tests were used to compare angular errors made in the first half of the training vs. those made in the second half of the training.

Furthermore, with the aim of exploring whether participants’ improvements were constant across the training days or whether instead they were focused on a specific day, we investigated daily participants’ performance, from days 2 to 5. We therefore performed a linear mixed model analysis including angular errors as the dependent variable, with day of training (Day) as fixed repeated-measure factor, plus random effects intercepts and slopes for Day. The model used Satterthwaite’s method for the estimation of degrees of freedom and the covariance structure for random effects was first-order autoregressive (AR1). *Post hoc* pairwise comparison *p*-values were corrected using Sidak’s method.

##### Tracking Exploration Strategies (Experiment 1)

Participants’ exploration strategies in the free exploration and in the object search phases were tracked. The specific sequence in which the bicycle components were found were recorded. In each day, we verified how many subjects found and collected the objects in the same vs. a different order. Furthermore, the time necessary to find the objects in the free navigation phase and in the object search phase was measured. Then, we averaged the time spent in exploration and in the object search, respectively, between days 2 and 3 (first half of the training) and between days 4 and 5 (second half of the training. Paired-samples *t*-tests were used to compare the time spent in searching the bicycle components in the first half of the training vs. in the second half of the training.

Furthermore, through paired-sample *t*-tests, we verified whether on a daily basis, the time spent to find the objects in the free navigation phase was different (possibly greater) than the time spent to find the same objects in the object search phase. Bonferroni correction was applied to *t*-test alpha level: 0.05/4 = 0.0125. Finally, with the aim of exploring whether participants’ exploration and object search times were constant across the training days, we investigated daily participants’ performance, from days 2 to 5, by performing the same linear mixed model analysis, exploited for the *Pointing task* (see above).

##### Spatial Memory Task

To evaluate subjects’ performance on the memory task, participants’ accuracy values were recorded and analyzed. Two subjects were excluded from subsequent analyses for a technical problem occurred during data recording. Paired-samples *t*-tests were used to compare participants’ accuracy values following the experimental condition (*MindTheCity!*) vs. following the control condition (Passive Navigation).

At the end of each testing session, we asked participants which strategies they employed to remember objects’ disposition. Importantly, all participants adopted the same strategies in both sessions (i.e., the employed strategies were the same following *MindtheCity!* and the Passive Navigation). The most common memorization strategies were based on color similarities between objects and on the spatial disposition of the objects in rows and columns. Some participants also tried to associate some objects according to their functions.

### Experiment 2 (Neuroimaging-Replicating Sample)

#### Experimental Procedures

Experiment 2 was conducted to explore possible brain changes associated to the use of *MindTheCity!*, thus representing a preliminary investigation of the possible neural networks involved in spatial learning strategies.

An independent group of other 23 participants performed the same training with *MindTheCity!* and the same pointing task as in Experiment 1 (notably in this preliminary experiment, none of the subjects performed the Passive navigation control condition). Furthermore, these participants underwent two fMRI sessions: one before the training (pre-training) and the other after the end of the training (post-training), thus to perform a preliminary investigation of the possible changes in neuronal activity imputable to the training.

Data acquisition was performed at the Molinette Hospital in Turin on a 3-T Philips Ingenia with a Sense high field high resolution head coil (MRIDC) optimized for functional imaging. Functional *T*_2_*-weighted images were acquired using a gradient-echo EPI sequence, with a repetition time (TR) of 2,800 ms and an echo time (TE) of 30 ms. The acquisition matrix was 96 × 96; the field of view (FoV) was 230 mm. For each paradigm, a total of 260 volumes were acquired. Each volume consisted of 31 axial slices, parallel to the anterior–posterior (AC–PC) commissure line and covering the whole brain; the slice thickness was 4 mm with a 0.5 mm gap. Two scans were added at the beginning of functional scanning and the data discarded to reach a steady-state magnetization before acquisition of the experimental data. In the same session, a set of three-dimensional high-resolution *T*_1_-weighted structural images was acquired for each participant. This data set was acquired using an Ultra-Fast Gradient Echo 3D sequence (3D TFE—Turbo Fast Echo for Philips scanners, equivalent to a MPRAGE sequence for Siemens scanners) with a repetition time (TR) of 11 ms, an echo time (TE) of 5 ms and a flip angle of 8°. The acquisition matrix was 384 × 384 × 229, 0.7 mm isotropic voxels.

The experiment was conducted with a block design paradigm, with 14 s of rest condition (counting task) alternating with 28 s of active condition (*Verbal memory task*). During the verbal memory task, subjects had to listen to 7 different pairs of abstract, semantically unrelated words. The same 7 pairs were repeated for the whole duration of the verbal memory task (for a total of 17 repetitions). Subjects were asked to memorize each word pair. Importantly, a completely different list of words was employed in the subsequent session of scanning. At the end of each session, outside the fMRI scan, participants were asked to recall the 7 word-pairs. Accuracy was recorded. For each session of scanning, subjects underwent two runs. Each run had an acquisition time of 12 min and 13 s.

#### Data Analyses

##### Pointing Task

These analyses fully replicated those employed in Experiment 1—Pointing task.

##### fMRI Analyses

fMRI data were analyzed with the BrainVoyager QX (Brain Innovation, Maastricht, Holland).

Imaging data were analyzed using Brain Voyager QX (BrainInnovation, Maastricht, Holland). Functional data of each subject were preprocessed as follows: (1) Mean intensity adjustment (2) Motion correction (3) Slice scan time correction (4) Spatial data smoothing with a Gaussian kernel with full width half maximum (FWHM) of 8 mm. (5) Temporal smoothing was performed to improve the signal-to-noise ratio by removing high-frequency fluctuations: a Gaussian kernel with full width half maximum (FWHM) of 2.8 s was used for this purpose.

After preprocessing, functional scans were coregistered with their 3D high-resolution structural scans. Then, structural MRI were transformed into Talairach space (Talairach and Tournoux, 1988): Finally, using the anatomical—functional coregistration matrix and the determined Talairach reference points, each subject’s functional time course was transformed into Talairach space.

The following procedure was performed for each task condition. A single design matrix was specified and the box-car time course of the design (Rest vs. *Verbal memory task*) was convolved with a predefined hemodynamic response function (HRF) to account for the hemodynamic delay (Boynton et al., 1996). The resulting time courses were entered into a General Linear Model (GLM) analysis to yield beta parameter estimates for subsequent group statistics.

Afterward, a second-level GLM analysis was performed on the group to yield functional activation maps of the pre-treatment and of the difference between post-treatment and pre-treatment (post > pre).

All statistical comparisons were made on z transformed scores and were computed at a statistical threshold of *p* < 0.05 corrected for multiple comparisons using false discovery rate correction (Benjamini and Hochberg, 1995).

Activated clusters were determined through automated routines in Brain Voyager and the statistical values for the local maxima of each region were calculated. Anatomical structures were labeled using the Talairach Daemon (Lancaster et al., 2000), a digitalized version of the Talairach atlas, available online at: https://bioimagesuiteweb.github.io/webapp/mni2tal.html. Regions included in each cluster were identified using a custom Matlab script based on the Talairach atlas and confirmed by visual inspection by a trained neurologist. fMRI images were created using Neuroelf v 1.1 rc2.

### Experiment 3

#### Experimental Procedures

Experiment 3 was conducted to measure the behavioral effects of *MindTheCity!* training on a word-pair memorization task (*verbal memory task*). All procedures replicated those employed in Experiment 1, except for what follows. Following the training with *MindTheCity!* and following the passive navigation control condition (with a 10-day break between conditions), participants were asked to perform a verbal memory task similar to the one specifically designed for Experiment 2, but with a shorter exposition phase, to increase task difficulty and avoid ceiling effects. The *Verbal memory task* consisted of a word-pair memorization and recall test. Participants were asked to memorize (duration of the memorization phase: 50 s) a list of 7 different pairs of abstract, semantically unrelated words (here we employed the same word lists used for Experiment 2). Importantly, following the training and the control condition, participants had to memorize a different list of words. After a distraction task (which replicated the procedures of Experiment 1), participants were then asked to recall the 7 word-pairs, one by one. Corrected responses and errors (intended as both missed elements and uncorrected associations) were recorded.

#### Data Analyses

##### Pointing Task

These analyses fully replicated those employed in Experiment 1—Pointing task.

##### Verbal Memory Task

To evaluate subjects’ performance on the memory task, participants’ accuracy values were recorded and analyzed by means of number of errors. A paired-samples *t*-test was used to compare participants’ accuracy values following the experimental condition (*MindTheCity!*) vs. following the control condition.

## Results

### Experiment 1 (Discovery Sample)

#### Pointing Task

Mean angular errors performed in the second half of training were significantly lower than those occurred in the first half of training (First Half: average ± *SD*: 146.76 ± 66.57 degrees; Second Half: average ± *SD*; 103.92 ± 42.10 degrees; *t*_22_ = 3.42, *p* = 0.001, *d*_*z*_ = 0.76; see also Table 1A).

**TABLE 1A:** Experiment 1—pointing task.

Average (°)	Day 2	Day 3	Day 4	Day 5
	
	68.69	78.08	51.37	55.10

Main effect of day	*F* = 6.489		*p* = 0.002	

Pairwise comparisons		Day 2 vs. Day 3 *p* = 0.745	Day 4 vs. Day 2 *p* = 0.032	Day 4 vs. Day 3 *p* = 0.003
	
		Day 4 vs. Day 5 *p* = 0.994		Day 5 vs. Day 3 *p* = 0.062

**FIGURE 2:**
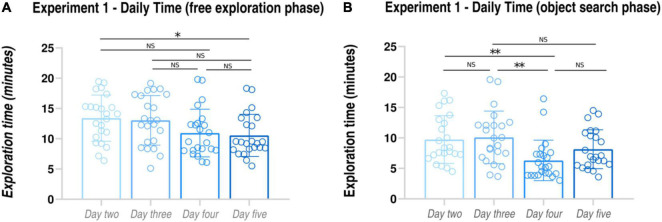
Tracking of navigational strategies in Experiment 1. *Daily performance in the Free exploration phase* **(A)**: subjects’ time needed to find the bicycle components. *Daily performance in the Object search phase* **(B)**: subjects’ time needed to find and collect the bicycle components. *p < 0.05; **p < 0.01.

Furthermore, participants’ daily performances (see Table 1A) were as follows: day 2 68.69 ± 35.36 degrees; day 3 78.08 ± 40.07 degrees; day 4 51.37 ± 25.31 degrees; day 5 55.10 ± 29.68 degrees (see Figure 2). Through the linear mixed model analysis, we found a significant effect of Day (*F* = 6.489, *p* = 0.002). *Post hoc* pairwise comparisons revealed that performances of day 4 were significantly improved as compared to day 2 (*p* = 0.032) and day 3 (*p* = 0.003). A marginally significant improvement was found between days 5 and 3 (*p* = 0.062). It is interesting to note that, despite the slight difficulty increase, no significant difference was found between days 2 and 3 (*p* = 0.745) and between days 4 and 5 (*p* = 0.994).

#### Tracking Exploration Strategies

On average, only 4 (± 1.41) out of 23 participants collected the bicycle components in the same sequence as the objects were found in the free exploration phase, thus indicating that the large majority of the subjects covered a different path between the exploration and the object search phase Furthermore, we averaged the time spent in the exploration and in the object search phases, respectively, between days 2 and 3 (first half of the training) and between days 4 and 5 (second half of the training). Paired-samples *t*-tests revealed that the time spent in searching the bicycle components in the first half of the training was significantly greater that in the second half of the training for both exploration and object search phases (free exploration phase: first half, 13.22 ± 3.22 min; second half, 10.75 ± 2.87 min, paired-samples *t*-test, *t* = 3.41, *p* < 0.001, *d*_*z*_ = 0.81; object search phase: first half, 9.91 ± 3.21 min; second half, 7.22 ± 2.79 min, paired-samples *t*-test, *t* = 4.78, *p* < 0.001, *d*_*z*_ = 0.89). Overall, these findings seem to indicate the adoption of a survey strategy to navigate the virtual environment, as suggested by the different covered paths between exploration and object search phases and by the shorter time needed to collect the bicycle components in the object search phase as compared to the exploration phase.

When looking at day-by-day performances (see Figure 2 and Table 1B), we observed that the time spent to find the objects in the free navigation phase was always significantly greater than the time spent to find the same objects in the object search phase (Day 2: free exploration, 13.40 ± 3.81 min; object search 9.73 ± 3.91; paired-samples *t*-test, *t* = 5.46, *p* < 0.001; Day 3: free exploration, 13.04 ± 4.09 min; object search 10.09 ± 4.30; paired-samples *t*-test, *t* = 3.06, *p* = 0.005; Day 4: free exploration, 10.95 ± 3.94 min; object search 6.31 ± 3.32; paired-samples *t*-test, *t* = 6.01, *p* < 0.001; Day 5: free exploration, 10.55 ± 3.48 min; object search 8.14 ± 3.19; paired-samples *t*-test, *t* = 3.22, *p* = 0.003). Finally, when comparing subject exploration performances on a daily basis, we found a significant effect of Day (*F* = 4.420, *p* = 0.011). *Post hoc* pairwise comparisons revealed that performances of day 5 were significantly improved as compared to day 2 (*p* = 0.026). Conversely, object search time results paralleled the daily accuracy pattern observed for the pointing task (see Figure 2), with a significant effect of Day (*F* = 9.747, *p* < 0.001). *Post hoc* pairwise comparisons revealed that the greatest improvement (expressed as a reduction of object search time) was observed between day 3 and day 4 (day 2 vs. day 3: *p* = 1; day 2 vs. day 4: *p* = 0.003; day 3 vs. day 4: *p* = 0.001; day 2 vs. day 5: *p* = 0.375; day 3 vs. day 5: *p* = 0.132; day 4 vs. day 5: *p* = 0.068). Notably, as for the pointing task, despite the slight difficulty increase, we did not find a significant difference between the performances of day 2 and day 3.

**TABLE 1B:** Experiment 1—tracking exploration strategies.

Free exploration average (min)	Day 2	Day 3	Day 4	Day 5
	
	13.40	13.04	10.95	10.55

Object research average (min)	Day 2	Day 3	Day 4	Day 5
	
	9.73	10.09	6.31	8.14

Main effect of day (Free exploration)	*F = 4.420*		*p = 0.011*	

Main effect of day (Object research)	*F = 9.747*		*p* < 0.001	

Paired samples *t*-tests	Day 2 *p* < 0.001	Day 3 *p* = 0.005	Day 4 *p* < 0.001	Day 5 *p* = 0.003

Pairwise comparisons	Day 2 vs. Day 3 *p* = 1		Day 2 vs. Day 4 *p* = 0.003	Day 2 vs. Day 5 *p* = 0.375
	Day 3 vs. Day 4 *p* = 0.001		Day 3 vs. Day 5 *p* = 0.132	Day 4 vs. Day 3 *p* = 0.068

#### Spatial Memory Task

Data analysis revealed that participants’ memorization performances were significantly enhanced following *Mindthecity!* training as compared to following the control condition (Mindthecity!: average ± *SD*: 28.76 ± 6.40; Passive Navigation: 25.81 ± 7.00; *t*_22_ = 2.60, *p* = 0.017, *d*_*z*_ = 0.45).

**FIGURE 3:**
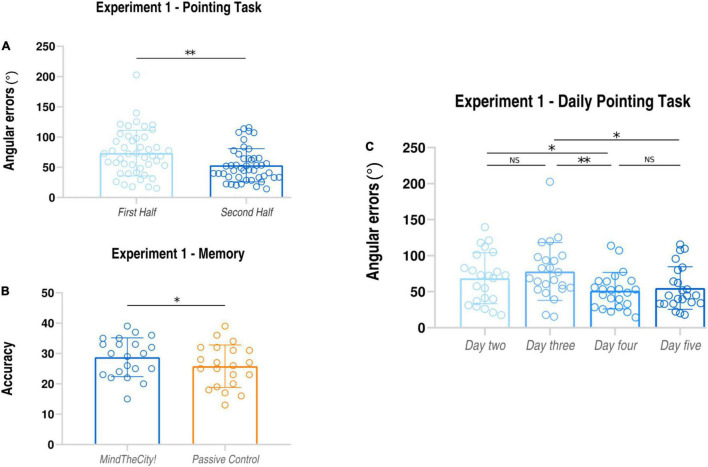
Results of Experiment 1. *Pointing task* **(A)**: subjects’ angular errors made in the first and in the second part of the MindTheCity! training. *Spatial memory task* **(B)**: subjects’ accuracy (absolute scores) following MindTheCity! and following Passive Navigation. *Daily performance at pointing task* **(C)**: subjects’ angular errors made in each day of MindTheCity! training. *p < 0.05; **p < 0.01.

Results of Experiment 1 are presented in Figure 3.

### Experiment 2 (Neuroimaging-Replicating Sample)

#### Pointing Task

**FIGURE 4:**
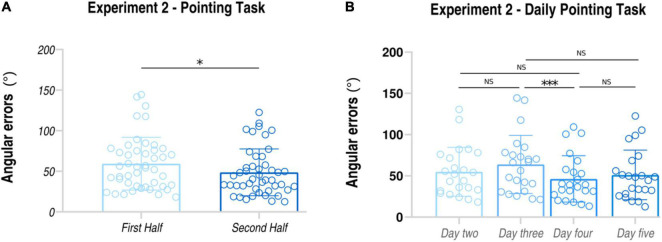
Behavioral results of Experiment 2. *Pointing task*: subjects’ angular errors made in the first and in the second part of the MindTheCity! training. *p < 0.05; ***p < 0.005.

Similarly to Experiment 1, the mean of angular errors made in the second half of the training was significantly lower than that in the first half of the training (First Half: average ± *SD*: 118.695 ± 54.303 degrees; Second Half: average ± *SD*; 97.434 ± 49.042 degrees; *t*_22_ = 2.29, *p* = 0.031, *d*_*z*_ = 0.46) (see also Figure 4). Importantly, behavioral data collected in Experiment 2 fully replicated the results of Experiment 1, thus confirming the validity of *MindTheCity!* training in improving subjects’ ability to map the virtual space in allocentric coordinates.

Furthermore, participants’ daily performances were as follows: day 2 54.85 ± 29.66 degrees; day 3 63.84 ± 35.18 degrees; day 4 46.29 ± 28.06 degrees; day 5 51.15 ± 30.06 degrees (see Figure 4 and Table 2). Through the linear mixed model analysis, we found a significant effect of Day (*F* = 3.544, *p* = 0.032). *Post hoc* pairwise comparisons revealed that performances of day 4 were significantly improved as compared to day 3 (*p* = 0.036). No other significance was found. Interestingly, despite the slight difficulty increase, no significant difference was found between day 2 and day 3 (*p* = 0.727) and between day 4 and day 5 (*p* = 0.963).

**TABLE 2:** Experiment 2—pointing task.

Average (°)	Day 2	Day 3	Day 4	Day 5
	
	54.85	63.84	46.29	51.15

Main effect of day	*F = 3.544*		*p* = 0.32	

Pairwise comparisons		Day 2 vs. Day 3 *p* = 0.727	Day 4 vs. Day 2 *p* = 0.729	Day 4 vs. Day 3 *p* = 0.036
	
		Day 4 vs. Day 5 *p* = 0.963		Day 5 vs. Day 3 *p* = 0.420

#### fMRI Activations: Post- vs. Pre-training and Verbal Memory Task

**FIGURE 5:**
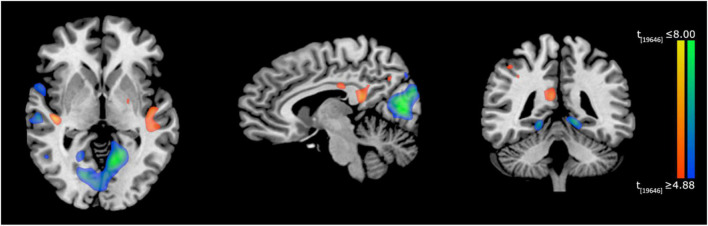
Neuroimaging results of Experiment 2. Activation differences (post minus pre-training) during the *Verbal memory task*, superimposed on a Tailarach brain template (TAL coordinates: *x* = –7, *y* = –49, *z* = 3), radiological convention. Red-to-yellow blobs indicate greater activations in the post-training task compared to pre-training, whereas blue-to-green blobs represent lower activations in the post-training task compared to pre-training.

With regard to fMRI analysis, neural activations during the *Verbal memory task* before and after training (post-training > pre-training) were compared. The results showed significant differences of activation in a spread network of brain areas, following MindTheCity! training. Specifically, post activations as compared to pre activations are shown in Table 3 (see also Figure 5).

**TABLE 3:** Post-training activations (compared to pre-training activations).

±		Brain region	Local maxima: x;y;z
Post +	RH	Superior temporal gyrus	58, –31, 9 *T* = 6.960; *p* < 0.001
Post +	RH	Putamen	23, 1, 11 *T* = 6.871; *p* < 0.001
Post +	LH	Inferior parietal lobule, including the supramarginal gyrus	–44, –38, 43 *T* = 6.394; *p* < 0.001
Post +	RH	Precuneus, including the inferior parietal lobule, the retrosplenial cortex and the posterior cingulate cortex (area 31)	13, –55, 34 *T* = 7.153; *p* < 0.001
Post +	LH	Precuneus, including the retrosplenial cortex and the posterior cingulate cortex (area 31)	–12, –58, 29 *T* = 7.545; *p* < 0.001
Post –	RH	Cuneus including bilateral Parahippocampal and Lingual gyri	3, –75, 16 *T* = –10.33; *p* < 0.001
Post –	LH	Superior temporal gyrus, including the enthorinal cortex	–51, 1, –8 *T* = –6.605; *p* < 0.001

Following both fMRI sessions, accuracy at the *Verbal memory task* were recorded. Participants’ accuracy was always at ceiling (with participants recalling on average 6 out of 7 word-pairs) and did not differed across sessions.

### Experiment 3

#### Pointing Task

Mean angular errors performed in the second half of training were significantly lower than those recorded in the first half of training (average ± *SD*, First Half: 249.56 ± 133.55 degrees; Second Half: 189.49 ± 119.90 degrees; *t*_13_ = 3.13 *p* = 0.004, *d*_*z*_ = 0.47; see also Table 4). Furthermore, participants’ daily performances were as follows: day 2 110.37 ± 82.94 degrees; day 3 139.18 ± 59.48 degrees; day 4 112.12 ± 69.06 degrees; day 5 77.35 ± 57.97 degrees (see Figure 5). Through the linear mixed model analysis, we found a significant effect of Day (*F* = 16.526, *p* < 0.001). *Post hoc* pairwise comparisons revealed that performances of day 5 were significantly improved as compared to day 3 (*p* < 0.001) and day 4 (*p* = 0.024). No other significance was found. Despite the slight difficulty increase, no significant difference was found between day 2 and day 3 (*p* = 0.248).

**TABLE 4:** Experiment 3—pointing task.

Average (°)	Day 2	Day 3	Day 4	Day 5
	
	110.37	139.18	112.12	77.35

Main effect of day	*F* = 16.526		*p* < 0.001	

Pairwise comparisons		Day 2 vs. Day 3 *p* = 0.248	Day 4 vs. Day 2 *p* = 1	Day 4 vs. Day 3 *p* = 0.134
	
		Day 4 vs. Day 5 *p* = 0.024		Day 5 vs. Day 3 *p* < 0.001

#### Verbal Memory Task

Data analysis revealed that participants’ memorization performances were significantly enhanced following *Mindthecity!* training as compared to following the control condition (number of correct responses, *Mindthecity!*: 4.43 ± 1.39; control: 3.43 ± 1.09; *t*_13_ = 2.87, *p* = 0.013, *d*_*z*_ = 0.79).

## Discussion

**FIGURE 6:**
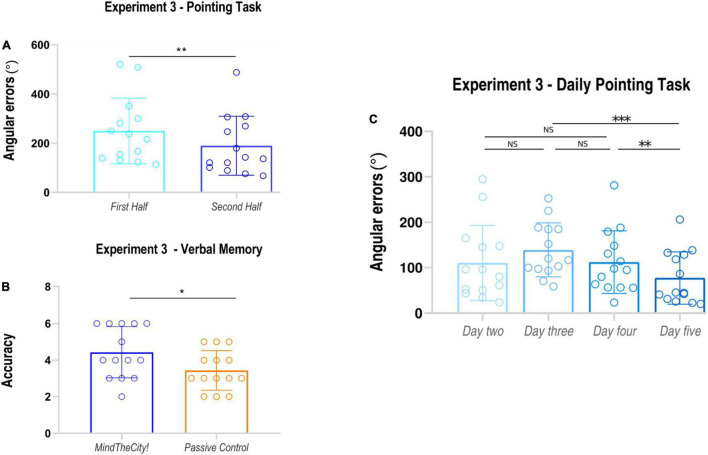
Results of Experiment 3. *Pointing task* **(A)**: subjects’ angular errors made in the first and in the second part of the MindTheCity! training. *Verbal memory task* **(B)**: participants’ accuracy (absolute scores) following MindTheCity! and following Passive Navigation. *Daily performance at pointing task* **(C)**: participants’ angular errors made in each day of MindTheCity! training. *p < 0.05; **p ≤ 0.01; ***p < 0.005.

Our findings showed that a virtual training with *MindTheCity!*, when compared with a passive navigation training, improve participants’ reorientation and visuo-spatial abilities (Experiment 1—Discovery sample, Figures 3, 4), while our preliminary neuroimaging results suggest a direct modulation of the brain activity in response to memorization tasks, even when not entailing the spatial domain (Experiment 2—Neuroimaging-replicating sample, Figure 5). The ability of *MindTheCity!* in enhancing memorization (even in a verbal domain) has been confirmed by the control Experiment 3 (Figure 6).

In the next few paragraphs, we will interpret our behavioral results (i.e., *Pointing task, Tracking exploration strategies*, and *Spatial memory task*) suggesting that a focused navigation in a virtual town might support the formation of allocentric maps, thus enhancing spatial memory encoding (section “*MindTheCity!* Training Enhances Allocentric Spatial Abilities: Behavioral Evidence”). Also, we will discuss our neuroimaging results suggesting the increase of activation of higher order brain circuits as a possible cause of enhancement of a general memory encoding mechanism, and the simultaneous decreased activation of the specialized memory circuits as cue of a reduction of the working load needed to perform the mnemonic task (section “Allocentric Spatial Abilities and Memory Performances: A Possible Link?).

### *MindTheCity!* Training Enhances Allocentric Spatial Abilities: Behavioral Evidence

In the present research, with the aim of testing the presence of behavioral modulations following *MindTheCity!* training, we exploited three different measurements (i.e., *Pointing task, Tracking of exploration strategies*, and *Spatial memory task*), all related to the ability to map the virtually navigated space. More specifically, in each session of the training (in Experiment 1, Experiment 2, and Experiment 3), we tested participants’ allocentric knowledge (Latini-Corazzini et al., 2010) of the virtual environment they were navigating, by asking them to point to different places within the virtual town (e.g., the starting point). Importantly, angular errors of such pointing task demonstrated that, in the second half of the training, participants’ performance was significantly improved as compared to the first half of the training. This finding is important since it demonstrates that participants’ ability to successfully map the virtual environment (necessary to perform the pointing task) was significantly enhanced throughout the training. Crucially, the improvement highlighted in Experiment 1 (our discovering sample) was fully confirmed in Experiment 2 (our replicating sample), which can be considered as a measure of internal replicability of the *Pointing task* results. Furthermore, similar results were also confirmed in Experiment 3.

Interestingly, results from the tracking of navigational strategies seem to indicate that most participants adopted a survey strategy to navigate the virtual environment, as suggested by the different covered paths between exploration and object search phases and by the significantly shorter time needed to collect the bicycle components in the object search phase as compared to the exploration phase. Since the employment of survey maps are considered related to the development of allocentric representations (Carelli et al., 2011), we believe that the present finding may represent supporting evidence of the ability of *MindTheCity!* training in promoting the space remapping in allocentric coordinates.

When looking at participants’ pointing task performances on a daily basis, we noticed that the greatest improvement was observed between days 3 and 4, in Experiments 1 and 2, and between days 3 and 5 in Experiment 3. This finding suggests that, overall, four sessions of training might be enough to improve participants’ spatial navigation abilities significantly. Importantly, despite the slight difficulty increase between days 4 and 5, performances never worsened between the two sessions. A similar pattern of results was obtained when observing participants’ daily performance at the object search task (see Figures 2, 3). This parallel between the participants’ performance at the two tasks might indicate that these two parameters might actually track the development of the same skill, possibly the ability to form allocentric representations (an aspect which seems to be crucial for the realization of both tasks).

Furthermore, results of the *Spatial memory task* (Experiment 1), by showing a significant difference following an active vs. a passive training, confirmed the efficacy of the *MindTheCity!* training in enhancing participants’ ability to build and remember the spatial relationships between different elements, independently from participants’ position in space. The *Spatial memory task* was crucial in demonstrating that this improvement in allocentric spatial encoding was not limited to the explored virtual environment but instead it may generalize to different tasks.

Overall, our behavioral results coherently demonstrated that a short training (1 h for 5 consecutive days) with *MindTheCity!* effectively potentiates young, healthy subjects’ ability to translate the spatial information acquired from an egocentric perspective to allocentric coordinates.

### Allocentric Spatial Abilities and Memory Performances: A Possible Link?

Favoring the construction of allocentric representations has a pivotal role in supporting spatial memory and navigation abilities. Interestingly, recent research demonstrated that the enhancement of spatial mnemonic skills may improve overall (i.e., even non-spatial) memory performances (Dresler et al., 2017). Therefore, to investigate whether the observed memorization enhancement might be generalized to other learning mechanisms (i.e., verbal memory), and to uncover the possible neural mechanisms underlying the effects of Experiment 1, we performed Experiment 2 and Experiment 3.

Overall our preliminary fMRI results, focused on the difference between *pre-* and *post-training* conditions, together with the behavioral results of Experiment 3, seem to indicate: (1) The involvement of less neural resources to achieve similar memorization performances, suggesting a more effective acquisition of the information to be learned; (2) the confirmation of Mindthecity! ability in improving the memorization of verbal information (Experiment 3); (3) the greater involvement of the right superior temporal gyrus and the bilateral retrosplenial cortex in the *post-training* condition, suggesting a possible shift toward visuo-spatial, imagery-based mnemonic strategies.

More specifically, in Experiment 2, after the *MindTheCity!* training, regions typically involved in memory processing—including parahippocampus, lingual gyrus and entorhinal cortex—decreased their activity. Deactivations in the memory circuits may constitute a correlate of training-induced less effortful processing. Accordingly, previous research interpreted the global activation decrease of the cerebral regions specifically involved in the training as a learning-related effect (Schiltz et al., 1999; Steele and Penhune, 2010; Howett et al., 2019). Interestingly, the parallel increase of the activity in the posterior cingulate cortex (area 31) *post* as compared to *pre-training* may be considered as a supporting evidence of this interpretation, since PCC greater activation has been often related to less attentionally demanding task (Leech and Sharp, 2014).

Furthermore, this specific pattern of memory-related areas deactivation paralleled with the increased activity of the regions involved in higher-level aspects of exploration and learning (such as the left supramarginal gyrus and the putamen) may also represent the neural mechanism underlying another function. A similar deactivation of the medial temporal lobe during learning has been previously found by Poldrack et al. (1999, 2001) who speculated that it may reflect an active suppression operated by other brain regions to improve performance. Hippocampal/parahippocampal regions work to retrieve declarative memories of specific previous trials: when performance does not rely upon specific episodic memories, suppression of that circuit would allow the rest of the network to work more efficiently (Mattfeld and Stark, 2015). In accordance with this view, we observed the greater activation of the left supramarginal gyrus, which is a part of the parietal memory network, contributing to both information encoding and retrieval (for a review see Gilmore et al., 2015), and the right putamen. Besides its recognized role in linguistic processing (as required by the present verbal memory task) (Viñas-Guasch and Wu, 2017), the putamen, as a part of the dorsal striatum of basal ganglia, has traditionally been associated with reinforcement learning (for a review, see Packard and Knowlton, 2002). Indeed, dorsal striatum play a critical role in processing response-contingent feedback and in maintaining information about reward outcomes (O’Doherty et al., 2004). In our *MindTheCity!*, retrieval of the bicycle’s parts constitute a rewarding outcome, giving direct feedback on the participants ability to map the virtual environment. Consistently, basal ganglia have been shown to be selectively involved in the ability to learn from explicit feedback, rather than implicitly (O’Doherty et al., 2004). Therefore, we can speculate that our training increased putamen activity as part of a brain network involved in the conscious construction of cognitive maps, based on the explicit feedback provided in the training. Interestingly, in their resting-state functional connectivity study, Di Martino et al. (2008) showed that right putamen seeds hold negative relationships with parahippocampal gyrus, and predict activity in areas linked to executive control. This is in line with the idea that putamen itself, besides being directly involved in high level aspects of cognition, might have had a role in balancing the activity of the medial temporal lobe and of the regions supporting higher-level, more general learning mechanisms (such as those involved in the present task).

Overall, we may interpret the post-training pattern of activations and deactivations as a result of a competing mechanism where regions involved in more general processes of memory enhance their activation to transfer the newly learned (visuo-spatial) mnemonic strategies to other learning domains.

When facing a memory task which is not related to the specific skills trained, such as the word association encoding we proposed during the fMRI scanning, broader memory abilities play a crucial role. It seems that our training, directed to the enhancement of spatial mnemonic skills, was also able to improve overall (i.e., even non-spatial) memory performances (as also demonstrated behaviorally by Experiment 3), by facilitating the acquisition of new information (as shown by the less effortful neural processing). This effect might also be due to the overlap in the neurocognitive systems responsible for word-pair memorization and visuo-spatial processing. Previous studies demonstrated the crucial role of parahippocampal and enthorinal regions in verbal memorization (see e.g., Goto et al., 2011; Jacobs et al., 2016; Liu et al., 2017). Therefore, it is possible that *MindTheCity!*, through its visuo-spatial training enhancing the processing of enthorinal and parahippocampal regions, concurrently improved spatial and verbal memorization.

Moreover, the increase in the activation of right superior temporal gyrus might indicate the greater deployment of visuo-spatial skills after the training. Previous research exploiting intraoperative electrical stimulation in awake patients during brain surgery showed that the right superior temporal gyrus has a crucial role in serial exploratory visual search (Gharabaghi et al., 2006). Other studies confirmed this finding. Ellison et al. (2004), using repetitive transcranial magnetic stimulation, demonstrated that the right superior temporal gyrus is pivotal in human exploration behavior. It is also involved in allocentric spatial processing (Shah-Basak et al., 2018) and spatial awareness (Karnath et al., 2001). Interestingly, we also found increased activity in the inferior parietal lobules and in the precuneus. This is an interesting finding, since it might again be related to the improvement of visuo-spatial abilities following the training. Studies on brain-damaged patients demonstrated that lesions of the parietal cortices, such as those related to hemineglect (i.e., a neural pathology where patients fail to detect and respond to sensory events occurring in the controlesional space; Vallar et al., 2003; Ronga et al., 2017a,b, 2019), are often involved in the detriment of object identification and localization abilities (Trés and Brucki, 2014; Vallar and Calzolari, 2018). Therefore, it is possible that the greater activation of the parietal cortices observed in the present study might be directly related to the improvement of participants’ spatial localization abilities.

This interpretation of the results, suggesting that the *MindTheCity!* training enhanced the employment of visuo-spatial skills as well as the ability to remap the egocentric spatial information in allocentric coordinates, is supported by the increased activation of the retrosplenial cortex *post* vs. *pre-training*. As demonstrated by previous studies, the retrosplenial cortex is the area specifically deputed to the shift between an egocentric and an allocentric spatial frame of reference (Vann et al., 2009) and its involvement is systematically observed when visuo-spatial mnemonic strategies are employed, both in super memorizers and control participants (Maguire and Frith, 2003; Dresler et al., 2017).

Overall our preliminary neuroimaging investigation seems to indicate that our training favored the enhancement of a general learning mechanism through an increase of activation of higher order brain circuits, and a simultaneous reduction of the working load on the specialized memory circuits necessary to perform the mnemonic task. Furthermore, the *MindTheCity!* training promoted the employment of visuo-spatial strategies as suggested by the increased activation of the right superior temporal gyrus, parietal cortices, and the retrosplenial cortex.

This study presents some limitations to the generalizability of the results. First of all, we recruited only male participants, thus hindering to predict the outcome of the present training for female participants. We recruited a sample of healthy, young men, and we did not perform a full neuropsychological assessment of visuo-spatial skills, omitting to explore whether some of the involved participants possessed exceptional spatial memory skills. Nonetheless, the absence of outliers in our assessment tasks suggests that this was not the case. Finally, the absence of a control task in the fMRI paradigm prevents the generalization of our neurophysiological results, which should be intended as a preliminary investigation. The present limitations should be addressed in future studies, which might further explore the possibility of *MindTheCity!* to improve visuo-spatial abilities as well as the duration of the observed effects.

Altogether, our behavioral and neurophysiological results showed that *Mindthecity*! is effective in promoting the employment of visuo-spatial skills and in supporting the encoding of allocentric space representations. These findings, if confirmed by future studies, may lead to several applications. Improving visuo-spatial skills in healthy subjects is crucial to enhance their ability to navigate space in everyday life. Furthermore, the *MindTheCity!* training, supporting the egocentric-to-allocentric representation encoding, might actively contrast the aging-driven physiological decline in navigation abilities (Klencklen et al., 2012; Bates and Wolbers, 2014). Concerning the clinical domain, our training might limit the pathological deterioration typical of the prodromal forms of dementia (such as the Mild Neurocognitive Disorder) and Alzheimer’s Disease (Pengas et al., 2010; Serino et al., 2014; Marková et al., 2015; Coughlan et al., 2019). Future studies should be directed to test whether *MindTheCity!* could be used as a possible clinical tool, intending to assess and possibly rehabilitate visuo-spatial impairments.

## Data Availability Statement

The raw data supporting the conclusions of this article will be made available by the authors, without undue reservation.

## Ethics Statement

The studies involving human participants were reviewed and approved by the Comitato di Bioetica di Ateneo—Università degli Studi di Torino. The patients/participants provided their written informed consent to participate in this study.

## Author Contributions

KS: conceptualization, methodology, formal analysis, writing – original draft and review and editing, supervision, project administration, and funding acquisition. IR: methodology, software, formal analysis, investigation, writing – review and editing, and visualization. PP: investigation and writing – review and editing. AC: methodology, software, formal analysis, investigation and writing – review and editing. ED: methodology, software, formal analysis, investigation, writing – review and editing. PS: methodology and writing – review and editing. GG: conceptualization, methodology, writing – review and editing, supervision and visualization. All authors contributed to the article and approved the submitted version.

## Conflict of Interest

The authors declare that the research was conducted in the absence of any commercial or financial relationships that could be construed as a potential conflict of interest.

## Publisher’s Note

All claims expressed in this article are solely those of the authors and do not necessarily represent those of their affiliated organizations, or those of the publisher, the editors and the reviewers. Any product that may be evaluated in this article, or claim that may be made by its manufacturer, is not guaranteed or endorsed by the publisher.

## Footnotes

1
https://unity3d.com/
